# ‘A System Only to Be Defended on the Principle of Positive and Ascertained Necessity’: Quarantine and Thomas Maitland’s Contribution to the Medical Debates of 1819 and 1824

**DOI:** 10.1093/shm/hkad091

**Published:** 2024-03-25

**Authors:** Evangelos (Aggelis) Zarokostas

**Affiliations:** University of Utrecht, Utrecht, Netherlands

**Keywords:** contagion debates, Ionian Islands, Mediterranean, imperial history, history of medicine

## Abstract

This article focusses on the ‘plague debates’ which took place in the British parliament in 1819 and 1824, where the opinions of non-medical experts were also taken into account; particularly those of officials who had acquired relevant practical experience. Such opinions were crucial in politicising the medical debate from one of the nature of plagues, towards an evaluation of the impact of quarantine more broadly. Paying closer attention at the correspondence between the colonial governor of Malta and the Ionian Islands, and the colonial secretary, it reveals a different aspect of the contagion inquiry in Britain—one considering medical knowledge about plagues that was highly speculative. While most historical works illuminate the establishment of what was considered to be medical evidence, there is less work about the political, economic or even personal motives which underlined interventions in these debates.

In February 1819, Sir John Jackson (1763–820), Member of Parliament for Dover and member of the Board of Directors of the East India Company, brought before Parliament a motion ‘that a Select Committee be appointed to consider of the Validity of the Doctrine of Contagion in the Plague; and to report their Observations thereupon to the House’.^1^ The motion was supported by Frederick Robinson, President of the Board of Trade, a few years after a wave of successive plague outbreaks devastated the populations of Malta and the Ionian Islands. The issue was brought before Parliament as a response to the provocations for the repeal of quarantine laws of the famous Charles Maclean.^2^ Whilst the Select Committee settled their immediate question over the contagious nature of the plague, a series of debates on quarantine reform continued for the next couple of years and evidence of non-medical experts but ‘men on the spot’ like the British governor of Malta and the Ionian Islands, Thomas Maitland, was examined as well. These contagion debates were widely publicised in newspapers and caused a sensation for both medical and non-medical experts.^3^

Historians have analysed the debates on the nature and the ways of transmission of the plague as part of an older clash between ‘contagionists’ and ‘anti-contagionists’, but mostly as a mere prologue of the ‘contagion debates’ that came after the Act of 1825, which brought reform and a certain liberalisation of the quarantine system.^4^ Viewed from a rather teleological perspective of the ‘triumphant progress of science’, there can be few ways to view what seems to be in retrospect a rather peculiar episode in the history of medical thought when two medical schools clashed fiercely over ‘touch’ and ‘air’.^5^ Such ideas on ways of transmission of the plague were superseded by the germ theory and the invention of the microscope which allowed the discovery of the bacillus *Yersinia pestis* that causes plague.^6^ Although often not analysed at length in scholarship, the enquiries of 1819 and 1824 offer insight not only into medical practice, but also into imperial history and the ways in which British officials consolidated imperial rule through medical reform and public health, particularly with regard to the new British dependencies of Malta and the Ionian Islands (acquired in 1815, in the aftermath of the Napoleonic Wars).^7^

Contemporary medical debates in the period under study should be of interest to the imperial historian as well, as medical writing and debate on plague and quarantine measures were also permeated by early-nineteenth century conservative ideas on colonial governance and the establishment of militarised colonial structures in the post Napoleonic period (more notably in the Mediterranean where British military presence was already large). The defeat of Napoleon in 1815 produced a new kind of conservativism when the empire became central to state formation in Britain and the metropole allowed a free rein to colonial governors.^8^ In this sense, epidemic outbreaks, revolutionary ‘threats’, far-reaching insubordinate populations, all could be presented as ‘facts’ from conservatives in order to push (and justify) for a significant prolonging of military rule and the implementation of extreme measures across the empire.^9^ The unusual status of the Ionian Islands as a protectorate (a nominally independent state but in reality ruled as a colony) and the turbulent political situation in the islands in particular, offer an excellent case of how a short-term epidemic crisis speeded up long-time political machinations, providing a free rein for the Colonial Office to set the terms of the constitutional status of the islands. In this sense, the parliamentary inquiries of 1819 and 1824 are a useful point of departure for the ways in which the idea of a supposed necessity of militarised rule over Mediterranean peoples permeated medical discourse. More to the point, as British presence in the Mediterranean is still under-represented in imperial historiography, the debates on contagion and quarantine can illuminate the British thrust towards continuing colonial presence in the Mediterranean; what is taken easily for granted in the post-1815 period.

The present article examines these debates in greater detail with two aims: firstly, to show how the manner in which contagionist principles were utilised by ‘men on the spot’ like Maitland in order to militarise the state’s responses against plague outbreaks across the British Mediterranean, and ultimately to consolidate colonial authority in the region. Secondly, it argues that such responses perpetuated, and were shaped by the particular circumstances and precedents of quarantine in the Mediterranean.^10^ For example, by criticising previous Venetian responses to epidemic outbreaks in the Ionian Islands (under Venetian rule from the mid-fourteenth century until 1797), the British sought to consolidate colonial rule in the islands, as well as situated themselves in opposition to Venetian structures of rule and health measures. On the other hand, reality on the ground and the immense cost on human lives and economies, forced the British to employ more pragmatic approaches, being unable to do away altogether the ‘ancient’ Venetian system. Taking into account the tensions between metropolitan impulses and pragmatism on the ground, the article responds to calls from historians Frederick Cooper and Laura Ann Stoler to study Britain and its colonies in a ‘single analytical field’.^11^ In the same sense, by bringing together periphery and metropole, the article looks at debates on contagion and quarantine reform and builds on a recent and growing literature which looks at the impact of the empire—and of Mediterranean quarantines in particular—on Britain’s public-health system.^12^

## The ‘Ancient System’ and the Ideological Dimensions of Quarantine Reform

After learning that a question relative to quarantine laws was brought before parliament, Maitland wrote to the colonial secretary, Earl of Bathurst, in April 1819: ‘I observe that Mr Robinson … seems to entertain doubt on the nature and character of those causes which lead to plague’.^13^ ‘This is a subject’, Maitland continued, ‘upon which, I possibly have had more occasion to pay a very deep attention, than any other individual in His Majesty’s service … having arrived at Malta in the middle of the plague, and having since then witnessed the beginning and end of three different plagues: one in the island of Gozo, one in the island of Corfu and one in the island of Cefalonia’.^14^ Maitland goes on to refer to the ‘treatment of plague under the ancient system’ as a system ‘of cruelty and tyranny’ but as also one ‘only to be defended on the principle of positive and ascertained necessity’.^15^ Maitland’s lengthy defence of ‘contagionists’ and of the necessity of quarantine circulated beyond the Colonial Office and was presented as evidence of a ‘new authority’ in a further committee in 1824 that followed the earlier one of 1819, and led to a relative ‘relaxation of quarantine laws’ without abolishing the laws of quarantine in their entirety nevertheless under the Quarantine Laws Bill of 1825 (a year after Maitland died). Maitland’s testimony was written in multiple copies and was published ‘*in extenso* in the newspapers’.^16^ Before considering the ‘contagion debates’ of the early-nineteenth century, it is imperative to examine the background of quarantine system in the Mediterranean.

To start with, this ‘ancient system’ that Maitland referred to was the legacy of Venetian rule in the eastern Mediterranean which the British inherited as a network of port-cities and quarantine stations stretching from the city of Venice across the Adriatic down to Crete and Cyprus (including of course, the Ionian Islands).^17^ When analysing the history of quarantine in the British Mediterranean even less acknowledged is the fact that it was during the Republic of Venice when this multipolar system was not only established but also governed.^18^ In terms of the supposed Ionian predisposition to ignorance and violence, as many travellers noted, the Venetian legacy on the islands was often criticised by many contemporaries for its ‘corruptive influence’. On the other hand, the public-health measures that the Venetians had established were more generally acknowledged for their utility, particularly with regard to their efficiency in a military-enforced isolation. The British essentially utilised the anti-plague measures of segregation and isolation—like maritime quarantine, or *cordon sanitaire*—as set up by Venetian authorities. These basic principles were followed also in various epidemic outbreaks, such as cholera or venereal diseases. The article will now turn to analyse the basic elements of the quarantine system under the Venetians as well as the, often-conflictive, ideological dimensions of quarantine in the British Mediterranean.

The ‘myth of Venice’ was an ambivalent inheritance to integrate into imperial ideology.^19^ In hindsight, British imperial attitudes towards the Venetian past seemed to reflect an awkward balance between condemning Venetian corruption and despotic rule on the one hand and praising the eloquence—and effectiveness—of public-health measures established in Venice and its dependencies. Few aspects of administration and disease-control resembled this more than the quarantine system and quarantine islands (called *lazaretto*),^20^ which were established in Venice in order to isolate those suspected of infection in times of plague.^21^ Forming an invisible barrier against epidemics, Venice took advantage of insular geography in the Mediterranean and a similar network of infrastructures was established across the *Stato da Màr* (Venice’s maritime possessions) during the years of the Serene Republic. For example, in the Greek islands and the mainland which were previously held under Venetian rule, one can track elements of *lazarettos* today. Medical theories that underlined Venetian anti-plague measures revolved around pestilential miasmas but in practice their efforts to contain the plague were quite efficient.^22^ Although lacking a scientific basis, anti-plague measures taken by the Venetians (and other Italian city-states like Florence or Genoa) formed a pioneering and institutionalised response to plague outbreaks, to be imitated by northern Europeans later on.^23^

The Venetians imported the policies of isolation and segregation as early as 1348 from the Republic of Ragusa (today’s Dubrovnik).^24^ A fact well established in historiography, the isolation of 40 days formed the foundations of the ‘ancient system’ of quarantine: incoming vessels were fumigated and scrubbed, the cargo they carried was aired and the crew and passengers were isolated and confined for a period of up to 40 days while being held under guard.^25^ Ultimately, adding to the fear of their own lives, quarantined newcomers had to go through the embarrassment of their personal property being searched, fumigated and aired, as well as facing the psychological consequences of temporary isolation from the outside world. Being economically reliant on local communities, institutions like the *lazarettos* had strict hierarchies and depended on a locally operated information network in times of sickness. During plague outbreaks, for instance, heads of households were instructed to report to the parish priest, who was in turn expected to send daily collated reports to the authorities regarding the numbers of sick and houses which were shut up, also noting the gender of those affected.^26^ Across Venice and her dependencies, local *lazarettos* were supervised by a Public Health Office (the *Provveditori alla Sanità*, founded in 1478).^27^ Paying particular attention to identifying cases of plague as early as possible, public-health officials were meticulously recording all deaths, symptoms and the length of illness.^28^

John Howard (1726–90), a philanthropist who travelled extensively in the Levant and collected his own evidence primarily on prison reform but also on other institutions like schools, hospitals or *lazarettos*.^29^ Howard compiled an account of *lazarettos* in Europe, explicitly writing about ‘wise and good’ regulations taking place in the *lazarettos* in Venetian times. By that time, however, there was ‘such remissness and corruption in executing these regulations, as to render the quarantine almost useless’.^30^ Howard’s calls were to improve, not to repeal quarantine altogether. Such calls for reforming quarantine legislation reflected an increase of complaints from merchants and travellers by the end of the eighteenth century and were given a new impetus in the period after the Napoleonic Wars.^31^ John Hennen (1779–828), an Irish-born military surgeon and a contemporary of British rule in the Ionian Islands (*Agglokratia* in Greek) described the quarantine establishment in the islands inherited by the Venetians as ‘a sink of corruption, dangerous to the health of the community, and a source of expense to the public’, where ‘reformation was most loudly called for in this department’.^32^ Medical topographies, which were published as memoirs of travelling physicians and surgeons, often highlighted the ideological dimensions of colonial presence and considered the British authority in the Mediterranean the main force for ‘improving’ the quarantines.^33^

The period between 1720 and 1780 and afterwards, further accentuated by the Napoleonic Wars of 1793–815, marked a shift across Britain and the empire towards a more systematic implementation of quarantine measures and legislation. A comparatively different system to other public-health measures, like the Ottoman Empire which did not prioritise quarantine of the sick officially.^34^ Ottoman medical approaches followed different strategies to contain epidemic disease from western Europeans.^35^ The consular presence and the proximity of British troops in the Ionian Islands, and nearby the Ottoman mainland, allowed for closer estimations of their measures, though these remained highly speculative and depended on personal observations.^36^ In Britain, this change towards systematisation was highlighted by preconceived Eurocentric notions about the inefficiency of the Ottoman system due to ‘Asian despotism’ which was not only evident in travel narratives during the period but also in the public debate, articulated in a redefined language of scientific and statistical reasoning of the early-nineteenth century.

The push for quarantine reform was parallel to calls for better organisation and medical innovations more broadly due to the high mortality rate of British troops during the wars with France. As argued elsewhere, the wars called for enquiries into the ‘Nature, and Causes of the Great Mortality among the Troops’.^37^ In reality, mortality caused by disease greatly superseded mortalities caused by trauma: in fact, in the course of the 22 years of the Napoleonic Wars, from 240,000 losses of British troops, only 13 per cent were caused by battle-related injuries.^38^ Campaigns like in the West Indies (1798) and the mortality rates of yellow fever caused great concern to contemporaries and a provided first-hand experience of the impact of epidemic outbreaks to British medical and military officials serving overseas. One such example was the 38-year-old brigadier-general Thomas Maitland who acquired his first experience dealing with an epidemic outbreak in the West Indies under the harsh conditions of the evacuation of Saint-Domingue in 1798. After the tactical abdication of the senior officer, Major-General John Whyte, Maitland succeeded him, and entered into negotiations with the famous rebel leader, Toussaint L’Ouverture. These negotiations led to the evacuation of Saint-Domingue, and Maitland’s insistence on carefully monitoring the casualties and the progression of the disease, established his capability as an administrator.^39^

## Different Approaches? The Plagues of Malta and Corfu

As many other colonial officials, Maitland was transformed, after a short political career as a Member of Parliament on three occasions, from a supporter of the Whig Charles Grey to an authoritarian and an outright proponent of British imperialism, nicknamed ‘King Tom’ (largely due to his draconian anti-plague measures).^40^ Maitland’s first great challenge in the Mediterranean would be the appearance of a devastating disease in Malta due to smuggled goods which made it from Alexandria through a merchant ship between 1813 and 1814.^41^ In the appearance of the plague all commerce and business seized as the city was locked down: ‘a city a few weeks before so flourishing and gay … was converted into a Lazaretto; and nothing was to be seen in it or heard but sights and sounds of woe’, an eye-witness wrote.^42^ When Maitland was appointed the governor of Malta and Gozo, he followed and enforced the measures of his predecessor, Sir Hildebrand Oakes, by establishing a *cordon sanitaire* and by subdividing towns into smaller districts, in order to detect ‘any attempt to evade the established orders’.^43^ Goods that were suspected of being contaminated were destroyed, while patients who were suspected of being infected were segregated.^44^ Cutting all communication and shutting up individuals and families in their homes in order to avoid social interactions and the spread of disease was the essence of government instruction to the public. From the outset, Maitland militarised anti-plague measures by utilising British troops to guard the cordons and took over the management of disease by holding his own counsel, bypassing the civil authority of the Health Committee; this brought him into conflict with army medical officers like Ralph Green.^45^ The plague ended in September 1814 and convinced the energetic governor of the necessity of segregation and isolation, but also of a more active role of the army under his command in cases of epidemic outbreak.^46^ To a great extent, his management of the plague gave enough incentive for the Colonial Office to appoint him a joint governor of Malta and the newly acquired British protectorate of the Ionian Islands (under the Treaty of Paris in November 1815) shortly afterwards.

Not even a month had gone by since the creation of this protectorate that a ‘malignant fever’ appeared in a district of seven thousand inhabitants in southern Corfu; seemingly in a similar way as in Malta: because of smuggled goods that had been brought in from the opposite shores of the Greek mainland.^47^ The deputy inspector of hospitals, John Tully, was accompanied by two Greek principal physicians (whose names do not appear in his book about the plague outbreaks, published years after his experience in the Mediterranean) and under their guidance reached the small village where disease first struck. After a short and ‘anxious investigation’, they discovered that the fever had actually broken out 1 month before: thirteen out of a population of 50 had already died.^48^ Tully, also a supporter of the ‘contagionist’ interpretation of plague outbreaks, realised that the disease ‘was then raging with considerable virulence’ rapidly spreading and causing panic in the same way as it had happened in Malta.^49^

James Charles Campbell, the civil commissioner of the Ionian Islands at the time, treated the ‘malady’ ‘with precisely the same precautions and measures as are adopted in cases of confirmed plague’.^50^ Campbell ordered the establishment of sanitary cordons with sentries across the southern part of the island and the village where the fever started was burnt after the removal of its inhabitants to a *lazaretto*, following Venetian procedures. Tully collected enough information to draw a map of the region where the plague started with the cordon lines, the exact names of the villages and the villages that were destroyed. He circulated, through the local *Gazette*, instructions to the public to prevent social interactions, as these would spread the disease. Breaches of the quarantine often occurred frequently when Ionian men and women escaped the confines of the quarantine to travel to the mainland. These individuals were condemned to death according to Venetian and French legislation on the breach of quarantine. Writing to Bathurst in January, Campbell informed him that the measures he took had produced ‘already the most successful results’. The practices of ‘separation, segregation, and expurgation … affords an infallible remedy against its baneful operations’.^51^ Tully, along with other officials, surveyed various villages approximately every 2 days, and gathered statistics on the number of patients and the dead. Their numbers were announced in ‘bulletins of the sick’ that were circulated throughout the islands.^52^ By February 1816, Campbell wrote to Bathurst that ‘the disease has been retained where it originally was discovered, but not before some examples of capital punishment were inflicted for notorious breaches of discipline and the sacred laws of quarantine’.^53^ The governor performed his duties, but in reality the disease kept spreading to the rest of the island of Corfu; this would become more obvious before long. Campbell would be soon released from his post and replaced by Maitland.

Maitland’s approach was different as he put less emphasis on investigating the causes of disease and less trust in local authorities and medical staff. His militaristic approach when managing the plague earned him the rather hagiographic characterisation as ‘a great human force controlled and driven by a will of iron’ in later Victorian depictions.^54^ Initially, he suggested to Bathurst the replacement of the troops who were stationed in the Ionian Islands since 1809 with the garrison in Malta. Maitland thought that the troops had formed ‘local connections with women’, ‘local habits’ and ‘local opinions about political questions’. ‘It is my wish’, Maitland wrote, ‘that we should at least start clear upon all these points, and that whatever is British there should take their feelings from the state in which things actually now stand – and not from what was formerly speculated upon – and I, therefore, think this measure one, if not of primary necessity, at least of fitness and expediency under the circumstances’.^55^ This constant ‘militarisation’ of British authorities towards the outbreaks in Malta and the Ionian Islands served a purpose as elsewhere in the empire in separating the British from their subjects (or from the ‘protected’ dependencies in the case of the Ionian Islands), for example India.^56^ In this sense, the management of the disease by the army was not situated merely within a context of protecting the local population, but also was promoted as a proof of their supposed inability to protect themselves.

In essence, the new approach was to transform the entire island into one *lazaretto* where different areas and villages were assigned different levels of ‘suspicion’ and for the authorities to apply attention accordingly. The healthy and the suspected of infection were geographically divided by large encampments that were established in the countryside.^57^ Two days upon Maitland’s arrival in Corfu, he formally replaced Campbell, and rushed to reorganise the efforts for the control of disease: he concentrated the pest establishments at a distance from the villages which were previously stationed in different places in order to bring the contagion into one spot and set up sentries all round.^58^ While rearranging the cordons he quickly became disillusioned with Campbell’s supposed ‘successful results’. Almost as soon as he arrived in Corfu, Maitland wrote to London that he ‘found that the plague unfortunately prevailed, and has not in the smallest degree been got under, in about a third of the island – the rest of the island is stated to be uncontaminated and I hope it may prove so’.^59^

However, there were other reasons for Campbell’s replacement besides of his management of the plague, as it was assumed that he was prone to be manipulated by his circle of Ionian collaborators. In private, Maitland blamed local ‘intrigues’ and Campbell’s ‘soft’ approach to local elites, and that they might have undermined quarantine regulations and might have threatened the spread of the plague further. Because of Maitland’s previous illustrious career in other colonial posts (i.e. Ceylon or Malta) and due to his dense network of connections in the metropole, his ideas were quickly endorsed by the Colonial Office. The new High Commissioner found his predecessor ‘perfectly well disposed, but certainly sore in an idea that the intrigue of the people of this island had had an effect to hurt his character in the opinion with those with whom he wished to stand best. And from this feeling he was extreme anxious to get away immediately’.^60^ It was exactly by this vague wording referring to Ionians in general, that Maitland convinced London that he was the only suitable for the job of managing the disease. Not only that, but also Maitland’s different, and more hardline approach to Ionians who sought to push their agendas, would pave the way for the consolidation of British rule on the islands. Two months later, and the Colonial Office gave Maitland ‘every power to act as the King’s’ commissioner’, as ‘no one knows so well how to meet and arrest this formidable evil’.^61^

Campbell died in June 1819. While no academic study has carefully compared Campbell’s and Maitland’s respective measures, it seems that Maitland’s cordons proved effective, and the plague ended by March 1816. According to Tully’s calculations, 375 people had died in approximately 4 months in the area of Lefkimmi alone.^62^ In reality though, Campbell never had the chance to testify in parliamentary debates as to whether he believed the policies of isolation were proof towards the contagionist principle; or [simply] as an eye-witness of the plague in Corfu. Maitland’s different approach—based on the greater use of the army and swift punishment in cases of quarantine violations—did not address the nature of the plague nor of its ways of transmission, but rather he considered Campbell’s precautionary measures too ‘loose’ (such as monitoring the disease and the cordons).^63^ In addition, Maitland wrote to Bathurst, the measures to eradicate the plague would require high expenditures which were ‘beyond the limited means of the revenues of the islands’ and to prevent a further increase of the disease.^64^

If anything, for Maitland the plague of 1815 in Corfu (and in Cephalonia later on) was a proof of the supposed necessity of British protection over the Ionians. According to him, Ionians were unable to protect themselves from epidemic outbreaks; a statement which totally disregarded the efficacy of the ‘ancient’ Venetian system or the ability of Greek physicians whose value was often acknowledged in medical topographies of British medical practitioners. ‘At the very outset’, Maitland continued, ‘we have the strongest practical instance of the inability of the Ionian Republic to carry on itself – and if the plague increases, I can have no doubt not only that they will apply to us, but that in fact aid must be given to them’.^65^ It was no mere coincidence that Maitland’s suggestions ranged across anti-plague measures, expenditure and the supposed inability of the Ionians to rule themselves; it was during this period when the British governor and the colonial secretary were debating how they would rule the islands constitutionally but by maintaining real power on British hands.^66^

## Militarisation, Quarantine and Colonial Rule

Maitland disapproved not only the ‘loose’ measures of his predecessor—or rather his propensity to fall ‘victim’ to Greek intrigues—but, like Malta, his entire medical staff. One can imagine his scolding would make anyone under his employment cower in shame. After all, he earned his nickname as ‘King Tom’ and his image as a grumpy, yet skilful authoritarian, firstly from his subordinates, and then from the Maltese and Greeks he ruled.^67^ Referring to ‘a young man of the name of Tully’ to the colonial undersecretary, Henry Bunbury, the commissioner wrote that the young surgeon was ‘completely uninformed of the common practical rules upon the occasion – active enough but sanguine and ignorant to a degree – in truth in the way he was going on he never could have got rid of it – nor will that be an easy job now’.^68^ Indeed, as Tully himself admitted 5 years later, he was ‘unschooled’ in ‘the system requisite for the eradication of plague’.^69^

There is a common feature across the medical topographies of three British medical officials that the present article has examined and which seems to be the point where medical expertise and military enforcement intersected; a growing distrust, as the plague progressed, over the local village authorities in Corfu.^70^ Not being able to conceal his contempt for individuals, the lower clergy and local authorities, who, seeming reluctant to share information due to the unusual state incursions into village communities (instead of parish priests), Tully blamed individuals who hid ‘under the shadow of religion’ and the devastating impact that this had on the spread of disease.^71^ Hennen, on the other hand, was even less moderate with the clergy, whom he considered as ‘tyrannical’, ‘ignorant’ and ‘superstitious’; ‘taken from the very scum of the population’.^72^ A familiar mantra amongst many European travellers (including of course British officials who travelled to Greece), Hennen claimed that the ‘Greek character’ was ‘debased by their long endurance of Turkish tyranny and Venetian prostitution, and ‘one of the principal causes is to be found in the depravity and ignorance of their clergy’.^73^ There were, of course, more subtle differences in the ways that Hennen and Tully depicted the ‘Greek character’: Hennen, for example was more interested to assess more holistically all potential factors that could had contributed to disease—from human actions to climate and geography—while Tully was more concerned with the particular cases of plague outbreaks in Malta and the Ionian Islands. Nevertheless, most medical officials, like the ones mentioned above, were equally hostile of religious superstition and skeptical of the grip that the Orthodox clergy held on the ‘ignorant’ Greek ‘lower orders’, especially in cases of violations of quarantine regulations on ‘seclusion and separation’, for religious purposes. Tully mentioned those as shameful ‘violations of the established laws ‘under the shadow of religion’.^74^ However—and it needs to be mentioned here—the racial views of medical officials like Tully and Hennen were situated in the popular monogenist notion until the mid-nineteenth century, that human characteristics were not biologically immutable but were rather shaped due to culture and otherwise ‘external’ factors.^75^ Considering that these ‘external’ factors in the case of the Ionian Islands were the ignorance imposed by Venetian despotism and the Orthodox clergy, then Ionians could, according to contemporaries, be ‘civilised’ by an imperial power like Britain.

Yet, such stereotypical approaches on Ionian people exaggerated a recurring and culturally specific notion about the Ionian ‘lower orders’ as unruly and rebellious (a view, which, needs to be emphasised, was also shared by many members of Ionian nobility and amongst western-educated Greek physicians), implying a grip of the superstitious Orthodox clergy over Greeks to an extent much greater of what it actually was. In the end, Tully claimed that the experience of these outbreaks showed that the ‘faithful execution of the duties of the subordinate classes employed, under the critical circumstances of plague’ was not ‘to be relied upon’. ‘Nothing’, Tully continued, ‘short of the jealous eye of authority, and the overawing presence of a military force, thrown up to every door in an infected town, could ever ensure safety, or guarantee the due fulfilment of those measures’.^76^ Therefore, violations of quarantine violations could be easily translated, not as individual instances of panic, but as cultural characteristics of a backward and insubordinate population (particularly in times of acute epidemic crisis when the shift was from medically treating an individual to medically treating a population). Beyond medical topographies, this intellectual jump was by no means a rare occurrence in official correspondence amongst British officials. Moreover, incidents of what seemed to be a culturally driven disregard of public hygiene were also frequently presented as such in respect of the armed forces in numerous occasions in India.^77^ At the same time, it needs to be mentioned here, the application of ‘strict’ quarantine measures was at times selective depending on social position, wealth or personal connections; certain individuals seemed to be enjoying special privileges by being allotted to more comfortable residences, including a distinct space from the lazaretto and even nice dinners with the British governor.^78^

The practical experience that British medical and military officials had acquired from the Mediterranean was thus politicised, the efficiency of British governors in the management of the plague—particularly of Maitland—was largely exaggerated, and the role of Greek physicians and medical practitioners was not always acknowledged. It was because of the cordon sanitaire that were early established and due to Maitland’s vigorous measures that ‘the islanders owed their salvation’, wrote an enthusiastic contagionist, Augustus Bozzi Granville.^79^ To be sure, vigorous measures of isolation and segregation certainly played an important role, but we know now that other factors also played a role on the recession of the epidemic; social or climatic: in spring and in warmer climates infection rates slowed down due to fewer people staying indoors (and thus less prone to stay in close contact).

## The Contagion Debates in the Aftermath of the Plagues in the Mediterranean

As mentioned above, the debate of 1819 while Maitland was still alive, was widely publicised in the newspapers showing how the discussion over quarantine legislation was a topic of general concern to medical and non-medical experts alike.^80^ Politically, it polarised the debate between contagionists and anti-contagionists even further, even though both of these concepts were actually little defined and were often inseparable from economic interests or political imperatives.^81^ Maclean, for example, is a typical case whose medical and philosophical views were incorporated. The experience he acquired while serving at a pest hospital in Constantinople—under the patronage of the Levant Company, amongst others—convinced him about the fault of contagionist views and the ‘evil of quarantine laws’.^82^ Ultimately, Maclean believed that plague ‘depends upon the state of the atmosphere, as connected with the change of seasons, and with circumstances of soil, buildings, climate, and modes of living and etc.’^83^ He was also very critical of the health measures that were taken by British authorities in order to contain the plague in the Mediterranean. Without explicitly mentioning Maitland, Maclean mocked the management of disease ‘at Malta, where it may be presumed that some sort of medical treatment was attempted’, referring to ‘official testimony collected in the island’, that, many died at the height of the plague of 1813; ‘at the rate of ninety in the hundred’.^84^ For established institutions like the Royal College of Physicians the original doctrine of contagion ‘was sound and untouched by subsequent experiments’.^85^ The British government and the Board of Trade sought a way to ensure that the ‘validity of the doctrine of contagion in the plague’ remained ‘unshaken’; Bathurst wrote to Maitland to furnish information on quarantine laws ‘conducive to the end which the Board of Trade have in view’.^86^

In his letter to Bathurst, Maitland wrote that ‘it is not my intention to enter into any theoretical or medical discussion either with regard to the character or nature of contagion or infection, but to the limit myself simply to facts … which …lead every person to concur with one in opinion, that the plague is only acquired by contact’.^87^ And since contagion was the cause of the plague, ‘the treatment which has hitherto been followed, is the only mode of arresting so dreadful a calamity’.^88^ Maitland was not someone who would take criticism well, so he made an explicit mention of Maclean’s work on plagues, where ‘there is not a single instance quote in it, which come to my knowledge that is not most strongly presented and most unfairly stated’.^89^ Precautionary measures, according to Maitland, were the ‘cure’. ‘The whole’ of Mr. Robinson’s statement in the parliament’, Maitland wrote, ‘solves itself into the one examining whether ‘the plague be acquired by infection or contagion’. In many publications, he continued, infection or contagion were not defined at all, questioning the validity of medical authorship altogether: ‘in some they are most strangely jumbled together, and in not a few, they are altered exactly as suited the argument of the individual at the moment.’^90^ In fact, his views on medical professionals and the evidential basis of the nature of plague questioned medical authorship about plagues altogether: ‘medical advice with regard to the preventive treatment of plague’, Maitland claimed, was not only of ‘no use’ but was ‘almost invariably attended with evil consequence’, as every medical practitioner with a ‘favorite theory’ and an aim to ‘reduce the plague under his theory’. Ultimately, he was convinced that a pragmatic approach ‘by measures recognised by experience, acted upon for centuries’ would be the only viable strategy to contain plagues; what a popular belief held as a direct ‘visitation from God’.^91^

The commissioner concluded his letter in the same pessimism that the devastating impact plagues brought: ‘we are just as much in the dark in respect to any cure of this terrible disease, as we were at the moment it broke out at Malta’.^92^ However, at the same time, the quarantine system needed some amendment for Maitland nevertheless, mostly in terms of the length of the quarantine and the economic disruption this brought. Writing to his confidant, William A’ Court, pondered whether A’ Court could discreetly the Neapolitan government, a traditional ally to Britain, ‘to take off all further restraint of quarantine from the island of Malta where not a vestige of plague has existed for upwards of fifteen months’.^93^

The management of plague outbreaks (particularly by Maitland) left a lasting impression amongst the Maltese and the Greeks. Where it is obvious that medical topographies and ideas about contagion converged with imperial interests is the emphasis that medical officials put on the inefficiencies of previous administrations, that is Venice. As mentioned previously, British officials went to great effort to differentiate the protectorate of the Ionian Islands—and therefore to justify their own presence in the islands—with all previous administrations, most often Venice and her ‘corruptive influence’. In fact, ‘liberating’ the islanders from Venetian corruption and ‘protecting’ them from external threats, like an epidemic outbreak, gave from the outset a *raison d’être* of the British presence also as a leading force for social reform. In terms of imperial rhetoric, health policy in Venetian times was a great point of difference: Hennen mentioned the quarantine establishment where, ‘previous to the islands coming under British government, reformation was most loudly called for in this department’.^94^ ‘The most absurd and nugatory regulations’, the surgeon continued, ‘were formerly in force, while the corruption of those who regulated, and the poverty of those who carried their orders into execution, were much more calculated to spread than to check the progress of contagious disease’.^95^ He proceed to give an example:

Under the former regime, persons who had to perform different periods of quarantine were mixed indiscriminately in the same place, so that it often happened that in the same apartments were individuals who had finished their quarantine, and were on the point of being restored to free communication with the community, and others who had just arrived from suspected ports, and had to undergo the whole period of foul quarantine; an arrangement contradictory to the rules not only of quarantine, but of common sense. The poverty of the guardians was so great as to expose them to every temptation, and, instead of being selected from respectable persons, they were generally picked from among the vilest characters in the community.^96^

When Maitland, Bathurst and other imperial officials who saw British presence in the Mediterranean as a ‘civilising’ force referred to alterations of the ‘ancient’ Venetian system not only for reasons of efficiency but also as a rationalisation of imperial power in the region. In doing so, the line of conduct of the Colonial Office—particularly after the parliamentary inquiries of 1819 and 1824—was to acquire funds, maximum rhetorical efficiency and legitimacy in the metropole by integrating alterations to the quarantine system in the Ionian Islands with the debates on social reform and the ‘Old Corruption’ which were underway in the metropole since at least the 1790s.^97^ Meanwhile, a recurring criticism over the supposed propensity of Venetian rulers in the Ionian Islands to ‘corruption’ (of the kind that Hennen wrote in the quote mentioned above) had already gained ground in the late-eighteenth and early-nineteenth centuries amongst many British politicians and officials transcending across ideologies, as well as Ionian reformers who initially saw British protection as an opportunity to ‘fix’ social and political inequalities. Situated in the medical debates of 1819 and 1824, quarantine reform became intertwined with social reform more broadly.^98^ At the same time, however, the selective disregard of British officials of the efficiency of the Venetian quarantine system—particularly in the post-Napoleonic era—proved that, often-conservative reform could share the same neglect towards local and historic institutions and be equally ‘ahistorical’; in the same way as conservatives accused their opponents of being.^99^ Combined with a general metropolitan indifference over Britain’s dependencies before the colonial reforms of the 1830s and a widely assumed ‘necessity’ of British protection in the Mediterranean as a solution to Venetian ‘wrongdoings’ in the period under study, Britain’s ‘triumph’ over the plague outbreaks was utilised as a force for reform both in the metropole and Britain’s Mediterranean dependencies.

## Conclusions

After surviving a plague outbreak many contemporaries wanted to do away with the ‘damned’st’ quarantine altogether, along with all the boredom, stress and physical jeopardy that accompanied it.^100^ The end of the Napoleonic Wars and the commercial boom in the Mediterranean gave new impetus to increasing complaints over isolation and economic disruption, as well as demands for the appeal of quarantine laws, whether this came from merchants, travellers or liberal reformers. The general tendency of the Board of Trade was to regulate the severity of quarantine laws which they considered ‘too rigid’ due to the significant disruptions to economic life and the doctrine of contagion was a more politically viable solution than anti-contagionist principles. Moreover, interesting was the wording in official correspondence and the efforts of the Board of Trade and the Colonial Office—using Maitland’s testimony more notably—to control the agenda of quarantine reform by pushing forward arguments about the ‘necessity’ of quarantine and selectively applying the historicity of ‘ancient system’ (on the one hand acknowledging the necessity of the Venetian quarantine, on the other disproving Venice’s ‘corruptive influence’ altogether). In doing so, ‘contagionist’ responses against the attacks of liberal anti-contagionists—like Maclean—to formal medical institutions, can be analysed *also* within a broader scope of conservative reaction towards liberal principles in the early-nineteenth century.

On the other hand, debates on medical reform worked rather differently in the Ionian Islands. After the plague in Corfu, the commissioner proclaimed measures to reform the quarantine system, mainly to fix the ‘sink of corruption’, as Hennen described it. Maitland divided the *lazarettos* into separate districts and raised the pay of the guardians so that they would be less inclined to accept bribes. Finally, as the guardians of the *lazaretto* were poor and ‘from among the vilest characters in the community’, their ‘characters’ were ‘most rigidly investigated before they [were] appointed to act’.^101^ Owing to Maitland’s efforts, Hennen wrote in his book, ‘the quarantine establishment’ was ‘placed upon a respectable and efficient footing’, yielding ‘a considerable revenue’ from a percentage on expurgated goods or tax on the individuals who stayed at the *lazaretto*. The establishment was, according to the medical official, ‘to the highest state of perfection’.^102^ When disease struck again in Cephalonia in 1816, the Ionian state under the ‘protection’ of Britain, would be better prepared to control the plague. Despite efforts to regulate the *lazarettos* and to minimise bribes, often the time to spend in quarantine for persons detained on board (and suspicious of infection) was still applied selectively: some spend the entire time of isolations while some were allowed to land immediately, with Maitland himself breaching quarantine regulations on multiple occasions.^103^ British officials took some genuine efforts to improve measures against epidemic outbreaks admittedly aiming to minimise the cost in human lives and commerce. But ultimately, while the efficiency and ‘necessity’ of quarantine against outbreaks was still debated throughout the nineteenth century, the argument which remained much less challenged instead, was the implementation of strict measures by the army; whether this meant harsh punishment of quarantine violations or large encampments. If anything, the Colonial Office and Maitland himself emerged triumphant of the plagues in Malta and the Ionian Islands, securing a greater consensus in both the British parliament and amongst the Ionian elites, and thus more politically able to consolidate British power with the constitution of 1817. As the rest of the history of British protection in the Ionian Islands would show, the preservation of public health, under whatever circumstances, would become a *raison d’être* for British rule, and for the British to supersede local authorities, and to regulate the ‘unwanted’, in the same way as an emergency.

## Footnotes

1
*Report from the Select Committee. Appointed to consider the validity of the Doctrine of Contagion in the Plague*, 14 June 1819, 1133–34.

2 Charles Maclean (1766–824), educated as a physician, entered the service of the East India Company in 1788 and in this capacity undertook voyages in the Ottoman Empire and India. He became a prominent anti-contagionist and opposed quarantine regulations that were endorsed by the Tory government in the early-nineteenth century. See: Catherine Kelly, ‘‘Not from the College, but Through the Public and the Legislature’: Charles Maclean and the Relocation of Medical Debate in the Early Nineteenth Century’, *Bulletin of the History of Medicine*, 2008, 82, 545–69.

3 Catherine Kelly, *War and the Militarization of British Army Medicine, 1793–1830* (London and New York: Routledge, 2011), 140–44.

4 Medical opinion was polarised in political terms in early-nineteenth-century Britain between the ‘contagionists’, who believed that plagues and infectious diseases in general were the result of contagion, and the ‘anti-contagionists’ (or proponents of the ‘miasmatic theory’), who blamed the soil, air and the environmental conditions overall. It was a common characteristic of works on ‘medical topographies’, including works on the Mediterranean, for authors to situate themselves somewhere among these, or to adhere to different variations of these theories. On the clash between contagionists and anti-contagionists see Mark Harrison, *Contagion: How Commerce Has Spread Disease* (New Haven: Yale University Press, 2012), 50–79, *passim*; Kelly, ‘Not from the College’; Christopher Hamlin, ‘Predisposing Causes and Public Health in Early Nineteenth-Century Medical Thought’, *Social History of Medicine*, 1992, 5, 43–70; Erwin Ackerknecht, ‘Anticontagionism Between 1821 and 1867’, *Bulletin of the History of Medicine*, 1948, 22, 562–93.

5 We know now that there are different types of plague: the bubonic plague is transmitted mainly through flea bites while the pneumonic plague can be transmitted—much more rapidly compared with the bubonic plague—by close contact from an infected person or an animal through air droplets (see Frank M. Snowden, *Epidemics and Society: From the Black Death to the Present* [New Haven: Yale University Press, 2019], 51).

6 Even though it would be an oversimplification to define an entire medical community based on the ‘anticontagionist’ or the ‘contagionist’ camp. On this, see Tom Crook, *Governing Systems: Modernity and the Making of Public Health in England, 1830–1910* (Oakland: University of California Press, 2016).

7 This neglect has been addressed more recently, notably in the works of Catherine Kelly, ‘Not from the College …’ and *War and the Militarization*, *passim.*

8 What C. A. Bayly defined as ‘proconsular despotism’: the ‘prevailing ethos of loyalism, royalism and aristocratic military virtue’ cultivated by most colonial governors in Canada, Australia, India and the eastern Mediterranean, which was widely compatible with ‘the aims and attitudes of the regimes of the new conservatism in Great Britain’. C. A. Bayly, *Imperial Meridian. The British Empire and the World, 1780–1830* (London, 1989), 194–95, 199.

9 Alex Chase-Levenson, *The Yellow Flag. Quarantine and the British Mediterranean World, 1780–1860* (Cambridge: Cambridge University Press, 2020), 198; Boyd Hilton, *A Mad, Bad, & Dangerous People? England 1783–1846* (Oxford: Oxford University Press, 2006), 338–39.

10 In this sense, the article takes into account a broader literature with regard to representations and colonial rule as a ‘domination of strangers’ which nevertheless focus mainly on India. See Jon E. Wilson, *The Domination of Strangers. Modern Governance in Eastern India, 1780–1835* (Cambridge: Palgrave Macmillan, 2008); Catherine Hall, ed., *Cultures of Empire: A Reader—Colonizers in Britain and the Empire of the Nineteenth and Twentieth Centuries* (Manchester, Manchester University Press, 2000). On the Ionian Islands, see: Maria Paschalidi, ‘Constructing Ionian Identities: The Ionian Islands in British Official Discourses; 1815–1864’ (unpublished PhD thesis, University College London, 2009).

11 Frederick Cooper and Ann Laura Stoler, eds, *Tensions of Empire: Colonial Cultures in a Bourgeois World* (Los Angeles: University of California Press, 1997), 4.

12 Chase-Levenson, *The Yellow Flag*; Josette Duncan, ‘Health, Dominion and the Mediterranean: Colonial Medicine in Nineteenth-Century Malta, Cyprus and the Ionian Islands’ (unpublished PhD thesis, University of Warwick, 2014).

13 The National Archives (henceforth TNA), Colonial Office records (henceforth CO) 136/12, Maitland to Bathurst, Corfu, 13 April 1819, 124a.

14
*Ibid*.

15
*Ibid.*, 124b.

16
*Fraser’s Magazine for Town and Country*, January to June 1853, 47, 80.

17 Surely, the Venetians were not the first to impose isolation in times of epidemic outbreaks, but the Republic of Venice (as well as other Italian city-states) became famous for their state-led, efficiency in enforcing the strategies of segregation and separation by using the army at great effect, as well as due to the sophisticated administration which ran quarantine stations. There is yet another reason why we know more about European quarantines, and is simply because there is much less documentation about strategies of isolation elsewhere, that is in Muslim empires or China. For example, see: Birsen Bulmus, *Plague, Quarantines and Geopolitics in the Ottoman Empire* (Edinburgh: Edinburgh University Press, 2012); Steven Feierman and John M. Janzen, eds, *The Social Basis of Health & Healing in Africa* (Berkeley and Los Angeles: University of California Press, 1992).

18 On this, see the excellent work by Alex Chase-Levenson, *The Yellow Flag*, 67–91.

19 On the impact of the ‘myth of Venice’ on British imperial ideology see: Maria Fusaro, ‘Representation in Practice: The Myth of Venice and the British Protectorate in the Ionian Islands (1801–1864)’, in Melissa Calaresu et al., eds, *Exploring Cultural History: Essays in Honour of Peter Burke* (London: Routledge, 2010); John Eglin, *Venice Transfigured: The Myth of Venice in British Culture, 1660–1797* (Basingstoke: Palgrave, 2001); J. G. A. Pocock, ‘The Machiavellian Moment Revisited: A Study in History and Ideology’, *Journal of Modern History*, 1981, 53, 49–72.

20 The etymology of the word is still debated. The *lazarettos* were essentially quarantine stations with different rooms where people suspected of plague were isolated and moved to different rooms according to the state of their health. Contrary to medieval leper colonies, which were death houses with no curative purpose, *lazarettos* were plague hospitals aiming for plague victims to recover with albeit limited therapies (see Snowden, *Epidemics and Society*, 77).

21 The first *lazaretto* was established in Venice in 1423 and in the Ionian Islands between 1588 and the beginning of the eighteenth century (see Katerina Konstantinidou et al., ‘Venetian Rule and Control of Plague Epidemics on the Ionian Islands During the 17th and 18th Centuries’, *Emerging Infectious Diseases*, January 2009, 15, 41.

22 Snowden, *Epidemics and Society*, 71.

23
*Ibid*., 69.

24 Kira L. S. Newman, ‘Shutt Up: Bubonic Plague and Quarantine in Early Modern England’, *Journal of Social History*, 2012, 45, 809; Also, for a comparison with plague control in England later, see Paul Slack, *The Impact of Plague in Tudor and Stuart England* (London-Boston: Routledge and Kegan Paul, 1985). On managing quarantine in the Republic of Ragusa, see Zlata Blažina Tomić and Vesna Blažina, *The Health Office and the Implementation of Quarantine in Dubrovnik, 1377–1533* (Montreal, New York: McGill-Queen’s University Press, 2015).

25 Snowden, *Epidemics and Society*, 71.

26 Jane L. Stevens Crawshaw, *Plague Hospitals. Public Health for the City in Early Modern Venice* (Surrey, 2012), 81.

27 William Eamon, ‘Science and Medicine in Early Modern Venice’, in Eric R. Dursteler, ed., *A Companion to Venetian History*, *1400–1797* (London: Brill, 2013), 726. I would also like to thank Nikos Kapodistrias for this.

28
*Ibid*.

29 John E. Ransom, ‘John Howard on Quarantine’, *Bulletin of the Institute of the History of Medicine*, 1938, 6, 111–16.

30 John Howard, *An Account of the Principal Lazarettos in Europe with Various Papers Relative to the Plague Together with Further Observations on Some Foreign Prisons and Hospitals and Additional Remarks on the Present State of Those in Great Britain and Ireland* (Warrington: William Eyres, 1789), 22.

31 Kelly, *War and the Militarization*, 1–10; Harrison, *Contagion*, 51.

32 John Hennen, *Sketches of the Medical Topography of the Mediterranean: Comprising an Account of Gibraltar, the Ionian Islands, and Malta; to Which Is Prefixed, A Sketch of a Plan for Memoirs on Medical Topography* (London, 1830), 201–2.

33 Although scholars have tended to exaggerate cultural differences in these medical topographies as inherently Orientalist and hegemonic while European knowledge of the empire was indeed more complex. This is not the case that is in the Ionian Islands where some medical topographers have acknowledged the significant medical knowledge and practical experience of Ionian physicians: Hennen, for example, mentions Carlo Botta’s, *Storia Naturale e Medico dell’ Isola di Corfu*, which described diseases which occurred in Corfu in 1797; *Nozioni Miscellane intorno a Corcira by Lazzaro Mordo* (1808), or Baron Theotoky’s *Des Illes Ioniennes* (1815), as well as a number of an otherwise knowledge-rich Greek medical community (Hennen, *Sketches*, 205–6). See also comparatively: Mark Harrison, ‘Differences of Degree: Representations of India in British Medical Topography, 1820–c. 1870’, in Nicolaas A. Rupke, ed., *Medical Geography in Historical Perspective (Medical History Supplement)* (London: The Wellcome Trust Centre for the History of Medicine at UCL, 2000), 51–69. On the British Mediterranean, see Duncan, ‘Health, Dominion and the Mediterranean’, especially 158–59; Also, Wendy Jepson, ‘Of Soil, Situation, and Salubrity: Medical Topography and Medical Officers in Early Nineteenth-Century British India’, *Historical Geography*, 2004, 32, 137–55.

34 Alex Chase-Levenson, *The Yellow Flag*, 11; Konstantinidou et al., ‘Venetian Rule and Control of Plague Epidemics’, 42; In comparison with the Ottoman Empire, see: Birsen Bulmuş, *Plague, Quarantines and Geopolitics in the Ottoman Empire* (Edinburgh: Edinburgh University Press, 2012).

35 At the same time, however, Andrew White explains how the ‘most enlightened Turks’, applied some ‘measures of restraint’ with success across the Ottoman Empire, especially in the territories under the jurisdiction of Ali Pasha of Ioannina or the Vizier of Grand Cairo (Andrew White’s, *A Treatise on the Plague: More Especially on the Police Management of That Disease*. (London: John Churchill, 1846), 77).

36 For example, after receiving information from the British consul at Smyrna the British consul in the Ionian Islands in 1812, Spiridon Foresti mentioned a very severe plague raging in the Greek mainland, whose ‘daily deaths were then reckoned at about 1,500’ (The University of Nottingham Archives, Papers of Lord William H. Cavendish Bentinck, Pw Jd 2401, Foresti to Bentinck, Zante 24 September 1812).

37 D. P. Geggus, *Slavery, War and Revolution: The British Occupation of Saint Domingue 1793–1798* (Oxford, 1982), 348; Hector McLean, *An Enquiry into the Nature, and Causes of the Great Mortality Among the Troops at St. Domingo: With Practical Remarks on the Fever of That Island; and Directions for the Conduct of Europeans on Their First Arrival in Warm Climates* (London, 1797).

38 Martin R. Howard, ‘Walcheren 1809: A Medical Catastrophe’, *British Medical Journal*, December 1999, 319, 1645.

39 H. M. Chichester rev. Roger T. Stearn, ‘Maitland, Sir Thomas (1760–1824)’, *Oxford Dictionary of National Biography* (henceforth ODNB) (Oxford, 2004), http://www.oxforddnb.com/view/article/17835?docPos=4 [accessed January 2022]. On the British expedition in Saint-Domingue, see Geggus, *Slavery, War and Revolution*.

40 From 1790 to 1796, from March 1802 to February 1805, and from October 1812 to July 1813 (Chichester, ‘Maitland, Sir Thomas (1760–1824)’, *ODNB*, http://www.oxforddnb.com/view/article/17835?docPos=4) [assessed January 2022].

41 The history of the plague in Malta by eye-witnesses: Arthur Brooke Faulkner, *A Treatise on the Plague, Designed to Prove It Contagious, from Facts, Collected During the Author’s Residence in Malta, When Visited by That Malady in 1813* (London, 1820); Robert Calvert, ‘An Account of the Origin and Progress of the Plague in the Island of Malta’, *Medico-Chirurgical Transactions*, 1815, 6, 1–64.

42 John Davy, *Notes and Observations on the Ionian Islands and Malta with Some Remarks on Constantinople and Turkey*, vol. 1 (London, 1841), 44.

43 J. D. Tully, *The History of Plague as It Has Lately Appeared in the Islands of Malta, Gozo, Corfu, Cephalonia etc.* (London, 1821), 32.

44 W. C. Dixon, *The Colonial Administrations of Sir Thomas Maitland* (New York: Augustus M. Kelley,1969 [1939]), 132.

45 W. Bonnici, ‘Inspector of Hospitals Ralph Green and the Plague in Malta in 1813’, *Journal of Royal Army Medical Corps*, 1998, 144, 40–45.

46 Dixon, *The Colonial Administrations*, 132.

47 In the small village of Marathia, in today’s Lefkimmi. TNA CO 136/3, no. 70, Campbell to Bathurst, Corfu, 31 December 1815, 143a.

48 Tully, *The History of Plague*, 88.

49
*Ibid.*, 87.

50 Underlined in the original, TNA CO 136/3, Campbell to Bathurst, Corfu, 31 December 1815, 144.

51 Underlined in the original, TNA CO 136/4, no. 74, Campbell to Bathurst, Corfu, 20 January 1816.

52 TNA CO 136/4, no. 1 enclosure, ‘Bulletin of the sick in the District of Lefthimo’, dated 8 January 1816.

53 TNA CO 136/4, Campbell to Bathurst, Corfu, 12 February 1816.

54 Quote in Walter Frewen Lord, *Sir Thomas Maitland. The Mastery of the Mediterranean* (New York, 1897), xiv.

55 TNA CO 136/5, Maitland to Bathurst, Malta, 6 January 1816.

56 Douglas Peers, ‘Colonial Knowledge and the Military in India, 1780–1860’, *The Journal of Imperial and Commonwealth History*, 33:2, 2005, 167.

57 White, *A treatise on the plague*, 184–92.

58
*Ibid.*, 187–88.

59 TNA CO 136/5, Maitland to Bunbury, Corfu, 18 February 1816, 13.

60
*Ibid*.

61 TNA CO 136/5, Bunbury to Maitland, Downing Street, 4 March 1816.

62 Tully, *The History of Plague*, 115.

63 TNA CO 136/5, Maitland to Bathurst, Corfu, 27 February 1816, 20.

64
*Ibid*.

65
*Ibid*.

66 The Constitution of 1817 (or Maitland’s constitution) has become a subject of an exhaustive, and often repetitive, debate in Greek and English historiography on the ‘peculiarities’ of the Ionian Protectorate being nominally an independent state which was nevertheless ruled as a colony in reality; thus it will not be analysed here. See Maria Paschalidi, ‘Constructing Ionian Identities: The Ionian Islands in British Official Discourses; 1815–1864’ (unpublished PhD thesis, University College London, 2009), 74–110; J. J. Tumelty, ‘The Ionian Islands Under British Administration, 1815–1864’ (unpublished PhD thesis, University of Cambridge, 1953), 25–66.

67 See for example, the interesting comparison between Maitland and Ali Pasha of Ioannina by his subordinate, Charles James Napier, where he describes the former as ‘insufferably rude, and abrupt’ yet extremely capable as a governor, contrary to the latter being ‘extremely civil’, but also ‘a mass of fraud’ (Charles James Napier, *The Colonies, Treating of Their Value Generally, of the Ionian Islands in Particular: The Importance of the Latter in War and Commerce* (London, 1833), 141–42).

68 TNA CO 136/5, Maitland to Bunbury, Corfu, 27 February 1816.

69 Tully, *The History of Plague*, 98.

70 Mostly the works of Tully’s *The History of Plague*, Hennen’s *Sketches* and White’s, *A Treatise on the Plague*.

71 Tully, *The History of Plague*, 127.

72 Hennen, *Sketches*, 184.

73
*Ibid*.

74 The young physician seemed genuinely horrified on the lack of public awareness with regard to the infectiousness of the plague when he referred to one of these cases in today’s Melikia in Corfu during Easter: ‘But what was my surprise, when I received private information that three of the principal churches in Melicchia, had been clandestinely opened on Easter Saturday night, and service performed in all three to crowded audiences. This breach of public order took place on the 20th of April, 2 days previous to the breaking out of the plague in Melicchia’ (Tully, *The History of Plague*, 127). Any similarity with such instances with Greece in the early stages of COVID-19 is of course entirely coincidental (Greek churches open for Epiphany despite coronavirus lockdown | Greece | The Guardian) [accessed October 2022].

75 Nancy Stepan, *The Idea of Race in Science: Great Britain, 1800–1960* (London, 1982).

76 Tully, *The History of Plague*, 108, 109.

77 Harrison, ‘Differences of Degree’, 67; Douglas M. Peers, ‘The Habitual Nobility of Being’: British Officers and the Social Construction of the Bengal Army in the Early Nineteenth Century’’, *Modern Asian Studies*, 25:3, 1991, 545–69.

78 John Chircop and Francisco Javier Martinez (eds), *Mediterranean Quarantines, 1750–1914. Space, Identity and Power* (Manchester: Manchester University Press), 269; and for a contemporary review, see for instance the account of Hugh Williams, who mentioned the ‘small adjoining house’ that was allotted for the residence of the traveller and his companions, as well as the dinner with Maitland that followed (*Travels in Italy, Greece, and the Ionian Islands in a Series of Letters … in Two Volumes*, II (Edinburgh, 1820), 154).

79 Augustus Bozzi Granville, *A letter to the right honorable F. Robinson, M.P. on the plague and contagion with reference to the quarantine laws: including the history of plague conveyed direct from the Levant to five European ports of the Mediterranean, within the last six years: and also a detailed account of the experiments made on the subject of contagion in plague …* (London, 1819), 50.

80 The Select Committee consisted of: sir John Jackson, Mr Boswell, Mr Frederick Robinson, Mr Fowell Buxton, Mr Henry Clive, Mr Cust, Mr Dawson, Mr Wilberforce, Mr Fazakerley, Mr Davies Gilbert, Mr Wallace, Mr Sandford Graham, Mr Hudson Gurney, Dr Phillimore, Mr Morritt, Sir Charles Monck, Mr Macqueen, Mr Fleming, Mr Legh, Mr Heygate and Mr Bennet (T. C. Hansard, *Parliamentary Debates*, New Series, 11 February 1819, 39, 426).

81 On the different definitions of the concept, see Margaret Pelling, ‘The Meaning of Contagion. Reproduction, Medicine and Metaphor’, in Alison Bashford and Claire Hooker, eds, *Contagion. Historical and Cultural Studies* (London: Routledge, 2001), 15–38.

82 Kelly, ‘Not from the College’, 545–69; Charles F. Mullett, ‘Economics and Medicine: Charles Maclean and Anticontagion in England’, *Osiris*, 1952, 10, 224–51; Charles Maclean, *Evils of Quarantine Laws, and Non-existence of Pestilential Contagion …* (London, 1824).

83 British Library (henceforth BL) Add MS 59265, Dropmore Papers, Charles Maclean to Granville, park Place, 14 February 1816, 99a.

84
*Ibid.*, 95b.

85 T. C. Hansard, *Parliamentary Debates*, New Series, 11 February 1819, 39, 426.

86 TNA CO 136/303, Bathurst to Maitland, Downing Street, 31 July 1819.

87 Underlined in original, TNA CO 136/12, Maitland to Bathurst, Corfu, 13 April 1819, 127a–b.

88
*Ibid*.

89
*Ibid*.

90
*Ibid*., 126b.

91
*Ibid.*, 127a–b.

92 TNA CO 136/12, Maitland to Bathurst, Corfu, 13 April 1819, 147.

93 BL Add MS 41529, Heytesbury Papers, Maitland to A’ Court, Naples, 18 July 1815, 3.

94 Hennen, *Sketches*, 201.

95
*Ibid.*, 201.

96
*Ibid.*, 201.

97 With Erwin Ackerknecht’s now classic article, ‘Anticontagionism Between 1821 and 1867’ being one of the earliest, and for many years, the only exception. See for example Arthur Burns and Joanna Innes, eds, *Rethinking the Age of Reform. Britain, 1780–1850* (Cambridge: Cambridge University Press, 2003). There are many mentions on ‘liberal’ or ‘conservative’ medical reform with regard to innovations and the medical profession but no explicit mentions to quarantine reform. Of course, the list of works on the ‘Age of Reform’ is vast; very indicatively: On ‘Old Corruption’ see Philip Harling, *The Waning of ‘Old Corruption’: The Politics of Economical Reform in Britain, 1779–1846* (Oxford: Clarendon Press, 1996); on social and political reform see Mark Philp’s *Reforming Ideas in Britain: Politics and Language in the Shadow of the French Revolution, 1789–1815* (Cambridge: Cambridge University Press, 2014).

98 Mark Harrison, *Contagion*, 51.

99 Ian A. Burney, ‘Medicine in the Age of Reform’, in Burns and Innes, eds, *Rethinking Reform*, 171–72. Also, a useful analysis of the political and social context of the medical profession in Britain is: L. S. Jacyna, *Philosophic Whigs. Medicine, Science and Citizenship in Edinburgh, 1789–1848* (London and New York: Routledge, 1994).

100 ‘Adieu, thou damned’st quarantine, that gave me fever, and the spleen!’ wrote Lord Byron in his poem ‘Farewell to Malta’ in *The Works of Lord Byron: With His Letters and Journals, and His Life by Thomas More, Volume XVII* (London: John Murray, 1835), 239.

101 Hennen, *Sketches*, 201–2.

102 Hennen, *Sketches*, 202.

103 T. C. Hansard, *Parliamentary Debates*, New Series, 10 July 1823, XII, 1528.

## Notes

Evangelos (Aggelis) Zarokostas is a historian of Modern Greek and British imperial history, educated at the University of Athens (BA) and the University of Bristol (MA, PhD).

