# Publisher's Note

**DOI:** 10.1093/shm/hkag044

**Published:** 2026-06-05

Two articles in the current Special Issue, Ailing Empires, were previously published in Vol. 37, Issue 2, and Vol. 38, Issue 1. They are as follows:

- Evangelos (Aggelis) Zarokostas, ‘A System Only to Be Defended on the Principle of Positive and Ascertained Necessity’: Quarantine and Thomas Maitland's Contribution to the Medical Debates of 1819 and 1824, *Social History of Medicine*, Volume 37, Issue 2, May 2024, Pages 411–428, https://doi.org/10.1093/shm/hkad091
- Sarah Irving, Aleppo Buttons and Sulphur Injections: The Politics of Science and Citation in Mandatory Palestine and Transjordan, *Social History of Medicine*, Volume 38, Issue 1, February 2025, Pages 1–18, https://doi.org/10.1093/shm/hkae033

The articles are linked here so that readers can view the full collection together when the Special Issue publishes online.

